# Genetic variability of the envelope gene of Type D simian retrovirus-2 (SRV-2) subtypes associated with SAIDS-related retroperitoneal fibromatosis in different macaque species

**DOI:** 10.1186/1743-422X-3-11

**Published:** 2006-03-06

**Authors:** Jeannette Philipp-Staheli, Taya Marquardt, Margaret E Thouless, A Gregory Bruce, Richard F Grant, Che-Chung Tsai, Timothy M Rose

**Affiliations:** 1 Department of Pathobiology, School of Public Health and Community Medicine, University of Washington, Seattle, Washington, USA; 2 Washington National Primate Research Center, University of Washington, Seattle, Washington, USA

## Abstract

**Background:**

D-type simian retrovirus-2 (SRV-2) causes an AIDS-like immune deficiency syndrome (SAIDS) in various macaque species. SAIDS is often accompanied by retroperitoneal fibromatosis (RF), an aggressive fibroproliferative disorder reminiscent of Kaposi's sarcoma in patients with HIV-induced AIDS. In order to determine the association of SRV-2 subtypes with SAIDS-RF, and study the evolution and transmission of SRV-2 in captive macaque populations, we have molecularly characterized the *env *gene of a number of SRV-2 isolates from different macaque species with and without RF.

**Results:**

We sequenced the *env *gene from eighteen SRV-2 isolates and performed sequence comparisons and phylogenetic analyses. Our studies revealed the presence of six distinct subtypes of SRV-2, three of which were associated with SAIDS-RF cases. We found no association between SRV-2 subtypes and a particular macaque species. Little sequence variation was detected in SRV-2 isolates from the same individual, even after many years of infection, or from macaques housed together or related by descent from a common infected parent. Seventy-two amino acid changes were identified, most occurring in the larger gp70 surface protein subunit. In contrast to the lentiviruses, none of the amino acid variations involved potential N-linked glycosylation sites. Structural analysis of a domain within the gp22/gp20 transmembrane subunit that was 100% conserved between SRV-2 subtypes, revealed strong similarities to a disulfide-bonded loop that is crucial for virus-cell fusion and is found in retroviruses and filoviruses.

**Conclusion:**

Our study suggests that separate introductions of at least six parental SRV-2 subtypes into the captive macaque populations in the U.S. have occurred with subsequent horizontal transfer between macaque species and primate centers. No specific association of a single SRV-2 subtype with SAIDS-RF was seen. The minimal genetic variability of the *env *gene within a subtype over time suggests that a strong degree of adaptation to its primate host has occurred during evolution of the virus.

## Background

Type D simian retroviruses (SRV) are *Betaretroviruses *which have been etiologically linked to a simian acquired immune deficiency syndrome (SAIDS) of varying severity in several Asian macaque species. SRV infections are found in wild-caught macaques and have been endemic in captive macaque populations in the National Primate Research Centers (NPRC) in the United States. To date, five macaque SRV serogroups have been identified. All of the Type D SRVs are genetically and serologically related to the original prototype, the Mason-Pfizer monkey virus (MPMV), which was isolated from breast tumor tissue of a rhesus macaque (*M. mulatta*) in 1970 [1]. MPMV belongs to the SRV-3 serogroup and has been completely sequenced [2]. The prototype SRV genomic structure consists of only four genes flanked by LTRs on the 3' and 5' ends: the *gag*,*prt*,*pol*, and *env *genes encode the viral core proteins, the viral protease, the reverse transcriptase/endonuclease/integrase, and the envelope glycoproteins, respectively.

The SRV-1 serotype was first identified in the early 1980's in endemic infections of rhesus macaques at the California NPRC [3] and in rhesus macaques, Taiwanese rock macaques (*M. cyclopis*) and cynomolgus macaques (*M. fascicularis*) at the New England NPRC [4]. A California isolate, D1/RHE/CA, was obtained from a rhesus macaque [5] and has been completely sequenced [6]. A New England isolate, D1/CYC/NE, was obtained from a Taiwanese rock macaque [7]. Restriction enzyme analysis indicated that all three macaque species infected with SRV-1 at the New England NPRC contained the same SRV-1 subtype, presumably from the introduction of the virus into the colony from a single event [8].

The SRV-2 serotype was identified in the early 1980's in endemic infections of pig-tailed macaques (*M. nemestrina*), cynomolgus macaques, and Japanese macaques (*M. fuscata*) at the Washington NPRC [9-11], and in rhesus [12] and Celebes black macaques (*Macaca nigra*) [13] at the Oregon NPRC. Sequence analysis of SRV-2 isolates from a Celebes black macaque (D2/CEL/OR) [14] and a rhesus macaque (D2/RHE/OR) [15,16], both from the Oregon NPRC, demonstrated the presence of distinct SRV-2 subtypes. Partial sequence analysis of the *env *gene of an additional SRV-2 isolate from a pig-tailed macaque from the Washington NPRC (D2/MNE/WA) revealed a close similarity to the D2/RHE/OR isolate [17].

Differences in pathogenicity have been reported for different isolates within SRV serotypes. Such differences seem to depend on the virus subtype and the macaque species of the infected host. The SRV-1 isolate D1/RHE/CA, for example, was significantly more pathogenic in rhesus macaques than the D1/CYC/NE isolate [18,19], and differences in cell tropisms as a possible cause for such varying pathogenicity have been identified [20,21]. The SRV-2 isolate, D2/CEL/OR, caused severe immunodeficiency in Celebes black macaques but did not cause any symptoms when transmitted to rhesus macaques [13]. The D2/RHE/OR SRV-2 isolate was associated with mild immunodeficiency disease in rhesus macaques but caused severe fatal immunodeficiency disease in Japanese macaques. Furthermore, a closely related variant, D2/RHE/OR/V1, isolated from another rhesus macaque in the same endemically infected colony, caused severe illness in rhesus macaques [15]. A total of seventeen amino acid differences was detected between the two SRV-2 variants of which ten were located in the *env *gene. It was speculated that amino acid differences in the *env *gene could affect virus tropism and play an important role in determining pathogenicity.

Epidemics of SRV-2 associated SAIDS in pig-tailed macaques at the Washington NPRC and Celebes black macaques at the Oregon NPRC in the late 1970's and early 1980's were associated with a peculiar fibroproliferative syndrome, histologically defined as retroperitoneal fibromatosis (RF). RF is characterized by the aggressive proliferation of vascular fibrous tissue subadjacent to the peritoneum covering the ileocecal junction and the associated mesenteric lymph nodes. Two forms of RF have been recognized: the localized form in which fibroproliferative lesions occur in multicentric isolated nodules and the progressive form in which fibromatosis extends throughout the abdominal cavity [9]. In some animals, the localized form occured subcutaneously (subcutaneous fibromatosis (SF)) rather than in the usual abdominal location [22]. Because of its multicentric nature and its vascular and fibroproliferative features in a setting of profound immunodeficiency, RF and SF bear strong resemblance to AIDS-related Kaposi's sarcoma (KS) in humans.

In 1994, a novel gammaherpesvirus, Kaposi's sarcoma-associated herpesvirus (KSHV), was identified in both classical KS (HIV-independent) and AIDS-KS (HIV-associated). Epidemiological studies have demonstrated that KSHV is the etiological agent of all forms of KS, although HIV and the associated immunodeficiency syndrome are believed to be important co-factors in AIDS-KS. We have previously identified the macaque homolog of KSHV, called RF-associated herpesvirus (RFHV), in SRV-2 associated RF lesions [23] suggesting that RFHV may play an etiologic role in SAIDS-RF. However, SRV-2 is highly associated with SAIDS-RF and SRV-2 DNA is present in RF tumor lesions [12], suggesting that SRV-2 infection and the resulting immunodeficiency syndrome may play an important co-factor role in the development of RF.

SRV-2 associated SAIDS-RF has been observed in a variety of macaque species, including pig-tailed, rhesus, cynomolgus, Japanese and Celebese black macaques at different NPRCs in the United States. Analysis of a number of SAIDS-RF cases at the Washington NPRC revealed the presence of a single SRV-2 variant (D2/MNE/WA) associated with the RF lesions in pig-tailed macaques [17]. However, the SRV-2 variant D2/CEL/OR was also associated with SAIDS-RF in Celebes black macaques at the Oregon NPRC [13]. Sequence comparisons revealed significant differences between the partial *env *sequence of the D2/MNE/WA and the corresponding sequence of D2/CEL/OR [17], suggesting that multiple SRV-2 subtypes could be associated with SAIDS-RF. However, the molecular make-up of SRV-2 isolates associated with the various SAIDS-RF cases in different macaque species at different NPRCs has not been examined.

Despite the identification of KSHV as the etiological agent of KS, much remains unknown regarding KSHV transmission, life cycle and pathogenesis, and the role of retrovirus infection and immunodeficiency in disease progression. This is in large part due to the lack of a relevant animal model. Our long-term goal is to develop a macaque model of AIDS-KS using the KSHV homolog, RFHV, as an etiological agent to induce RF. Although it appears that SRV-2 plays an important role in the development of RF, it is not clear whether there is an optimal pathogenic SRV-2 subtype for disease induction. In this study, we have amplified and sequenced the complete SRV-2 *env *genes from four different species of macaques, with and without RF, from multiple NPRCs and the wild. We present here a detailed comparative sequence analysis of the different isolates and analyze their association with SAIDS-RF. We further examine the possible biological impact of sequence variation between isolates with respect to the functional domains of the envelope glycoprotein.

## Results

### Amplification, cloning and sequence analysis of the complete *env *genes of a wide variety of SRV-2 isolates

We have collected a number of RF tumor and non-tumor samples from different SRV-2 infected macaque species from a variety of sources, including captive macaques from six different US primate research centers and wild-caught animals from Singapore (Table 1). The tissue samples ranged from freshly frozen tissue from recent necropsies to 20–30 year old formalin-fixed paraffin-embedded tissue sections. Genomic DNA was isolated and used in PCR amplification to obtain full-length nucleotide sequences of the SRV-2 *env *genes. In some cases, sample amount and degradation limited our ability to obtain the full length sequence. We have analyzed the deduced amino acid sequences from these isolates and have compared them with the complete *env *gene sequences previously identified for an SRV-1 isolate from a rhesus macaque from the California NPRC (D1/RHE/CA; [6]), an SRV-3 isolate from a rhesus macaque from the Wisconsin NPRC (D3/RHE/WI; [2]), an SRV-2 isolate from a Celebes black macaque (D2/CEL/OR; [14]), and two closely related SRV-2 isolates from rhesus macaques (D2/RHE/OR and D2/RHE/OR/V1; [15,16]) from the Oregon NPRC. Additional partial *env *gene sequences previously identified from SRV-2 isolates of pig-tailed macaques at the Washington NPRC (D2/MNE/WA) [17] were also included in the comparison.

### Identification of six molecular subtypes of SRV-2 in captive and wild-caught macaque species by phylogenetic analysis of *env *gene sequences

**Figure 1:**
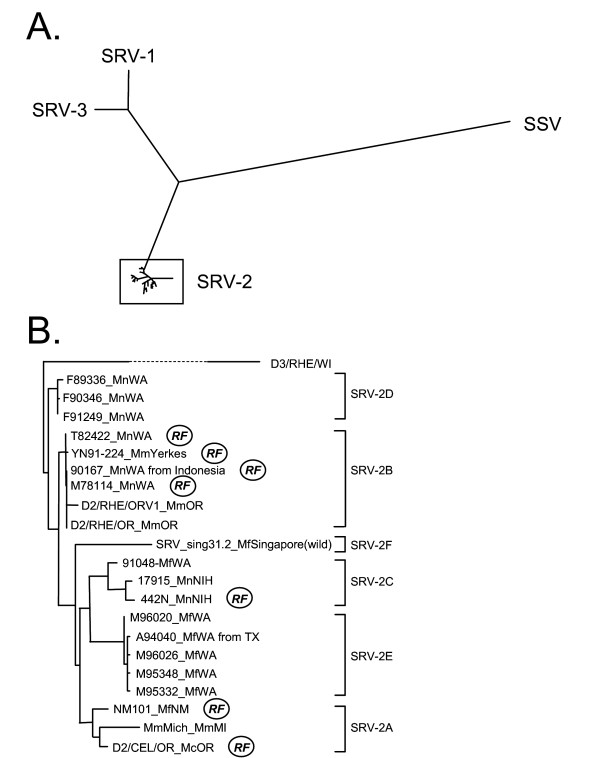
**Phylogenetic analysis of *env *sequences from different SRV-2 isolates**. (A) A phylogenetic tree of reference SRV-1, SRV-2 and SRV-3 *env *protein sequences and sequences obtained from the SRV-2 isolates in this study (see Table 1) was generated from a ClustalW multiple alignment using the protein maximum-likelihood method as implemented in the Phylip package (v. 3.62). The sequence of the distantly related simian sarcoma virus (SSV) *env *protein was used as outgroup [Genbank:NC001514]. (B) A detailed phylogenetic tree of the SRV-2 reference and isolate sequences was similarly generated using SRV-3 as outgroup. Emerging clusters were labelled as subtypes SRV-2A through 2F and virus isolates from animals diagnosed with RF are indicated.

We determined the complete sequence of the *env *gene from sixteen different SRV-2 isolates and the sequence of the C-terminal half of the *env *gene for an additional two SRV-2 isolates. The resulting eighteen *env *gene sequences were multiply aligned with the SRV-1, SRV-2, and SRV-3 prototype sequences obtained from the NCBI sequence database (Genbank), as indicated above. Using the distantly related simian sarcoma virus (SSV) *env *gene sequence as outgroup, we performed a phylogenetic analysis using the protein maximum-likelihood method. All of the putative SRV-2 sequences amplified from our tissue samples clustered closely together and were clearly distinct from the SRV-1 and SRV-3 prototype sequences (Figure 1A).

A closer phylogenetic analysis of the SRV-2 sequences revealed the presence of six separate clusters of sequences (Figure 1B). These clusters represent six molecular subtypes of SRV-2. The subtype SRV-2A cluster included the original SRV-2 prototype, D2/CEL/OR, isolated from a Celebes black macaque with SAIDS-RF at the Oregon NPRC in 1985 [13], a closely related isolate obtained in early 2000 from a cynomolgus macaque (NM101) with SAIDS-RF from the Lovelace Respiratory Research Institute in New Mexico, and a more distantly related virus, MmMich, obtained in the late1990's from a rhesus macaque at the primate center at the University of Michigan (see Table 1 for a description of viruses and their macaque hosts). The SRV-2B cluster included the previously characterized closely-related SRV-2 isolates obtained in the late 1980's from the rhesus macaque colony at the Oregon NPRC, D2/RHE/OR and D2/RHE/OR/V1 [15]. In addition, this cluster contained isolates obtained in the early 1980's from two colony-born pigtailed macaques (M78114, T82422) at the Washington NPRC, an isolate obtained in 1995 from a pig-tailed macaque transferred from Indonesia to the Washington NPRC (90167), and an isolate obtained in the mid 1990's from a rhesus macaque (YN91-224) from the Yerkes NPRC, which had all been diagnosed with SAIDS-RF. Also included in this subtype were a number of isolates from additional pig-tailed macaques with SAIDS-RF from the WaNPRC for which only partial sequences have been obtained (D2/MNE/WA) [17]. The SRV-2C subtype contained a previously unknown isolate from a pig-tailed macaque (442N) housed at the NIH primate center which had been diagnosed with SAIDS-RF in 1996 [24]. In addition, SRV-2 virus obtained in the mid-1990's from another pig-tailed macaque (17915) from the NIH, and from a cynomolgus macaque (91048) from the Washington NPRC, both without RF, contained similar sequences and grouped within the SRV-2C subtype. The SRV-2D subtype consisted of three virtually identical isolates obtained in the early 1990's from closely-related healthy pig-tailed macaques (F89336, F90346, and F91249) at the Washington NPRC. The SRV-2E subtype included isolates obtained from five closely related cynomolgus macaques at the Washington NPRC. Finally, the SRV-2F subtype consisted of an isolate obtained in 2003 from a cynomolgus macaque which had been sampled in the wild on the island of Singapore. Closely related isolates were identified in other cynomolgus macaques from the same geographical area (Richard Grant, unpublished data).

### Genetic variation of the *env *gene within SRV-2 subtypes

**Figure 2:**
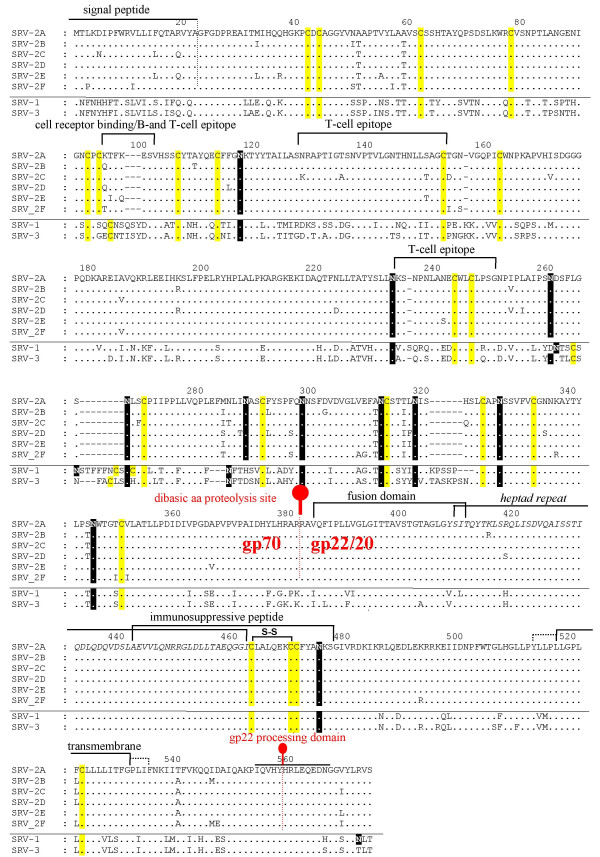
**Multiple alignment of the complete *env *sequences of representative prototypes of the SRV-2 subtypes**. A ClustalW alignment was generated using one representative prototype member from each of the six SRV-2 subtype clusters: SRV-2A (D2/CEL/OR), SRV-2B (D2/RHE/ORV1), SRV-2C (442N), SRV-2D (F90346), SRV-2E (A94040), SRV-2F (SRV_sing31.2). The sequences of SRV-1 and SRV-3 were included for comparison. Dots represent amino acids identical to the reference sequence of the SRV-2A prototype. Conserved cysteine residues are shaded in yellow, while putative N-linked glycosylation sites (NXT/S) are shaded in black. The putative signal peptide, known T- and B-cell epitopes, heptad repeat, as well as the gp20 fusion and transmembrane domains are indicated and referenced in the text. A predicted disulfide linkage within the immunosuppressive peptide, and the proteolytic cleaveage sites generating the gp70 surface and gp20 transmembrane subunits are indicated. While B- and T-cell epitopes have been determined for SRV-2, the functions and locations of other domains are derived from studies in SRV-3.

An alignment of the complete *env *sequence from prototypes of each of the SRV-2 subtypes revealed identical sizes (574 amino acids) and a high degree of conservation throughout the entire protein (Figure 2). The genetic variation between the *env *genes from isolates within a specific SRV-2 subtype was relatively small, with amino acid identities ranging from 97.3–100% (Table 2). In some cases, few, if any, amino acid differences were detected between the different isolates. This was true for the SRV-2F isolates from an endemically infected group of wild macaques in the same geographical area on the island of Singapore (unpublished data, R. Grant) and for the SRV-2D and SRV-2E subtypes where the different isolates came from a single primate center during the same time period and often consisted of macaques which were related through the dame or sire. The SRV-2E subtype included an isolate from A94040, a cynomolgus macaque that came to the Washington NPRC from Texas and was SRV-2 positive at that time, as well as isolates from descendents or siblings of descendents sharing the same sire. The isolates from A94040 and her child, M96026, were identical in sequence, while only 1–3 amino differences were observed with isolates from her child's half-siblings, M95348 and M96020 who shared the same sire. The SRV-2D subtype isolates from pig-tailed macaques F91249 and F90346 were identical and varied at only one amino acid position from F89336, which shared the same sire. While the isolates from F91249 and F90346 were amplified directly from peripheral blood leukocytes, the isolate from F89336 was obtained from an uncloned Raji cell tissue co-culture which had been maintained in A549 cells since 1996. Isolate F89336 serves as a reference strain within the Washington NPRC.

In the other subtypes, isolates obtained from different primate centers, from different macaque species and at different time periods were remarkably similar. In subtype SRV-2A, the original SRV-2 prototype, D2/CEL/OR, isolated by Raji cell co-culture of PBMC from a Celebes black macaque at the Oregon NPRC in the early 1980's, had only eight amino acid differences with an isolate (NM101) obtained 20 years later from a tissue sample of a cynomolgus macaque from the Lovelace Respiratory Research Institute in New Mexico. An additional SRV-2A isolate was obtained in the mid 1990's from a tissue sample of a rhesus macaque in a primate center at the University of Michigan. Although only the C-terminal half of this sequence was obtained, significant similarity with the other two SRV-2A isolates was noted. In subtype SRV-2B, two isolates (M78114, T82422) from the early 1980's, sequenced directly from PBMCs of colony-born pig-tailed macaques at the Washington NPRC, were identical in sequence. A third isolate (90167) obtained from PBMCs of a pig-tailed macaque at the same site ten years later varied by only one amino acid. This later macaque was captured in Indonesia and transferred to the Washington NPRC, suggesting that it became infected with the SRV-2B subtype already present in the colony. An SRV-2B isolate obtained from RF tissue of a rhesus macaque at the Yerkes NPRC in 1991 had only one amino acid difference compared to the M78114 and T82422 isolates from pig-tailed macaques obtained in 1984 at the Washington NPRC. The SRV-2B prototype, D2/RHE/OR, and the closely related D2/RHE/OR/V1, which were obtained by Raji-cell co-culture from PBMCs, contained four and nine amino acid differences, respectively, with the Washington isolates. Two closely-related SRV-2D isolates were obtained from the NIH primate center in Bethesda, MD. The SRV-2D prototype was obtained in 1996 from RF tissue of pig-tailed macaque 442N while the 17915 isolate was obtained from PBMCs from another NIH pig-tailed macaque. These two sequences varied at four amino acid positions. A partial *env *sequence was obtained in 1997 from PBMCs from a cynomolgus macaque (91048) which had been transferred to the Washington NPRC from Indonesia. This sequence varied from the 442N prototype by five amino acids within the c-terminal half.

### Genetic variation of the *env *gene between different SRV-2 subtypes

**Figure 3:**
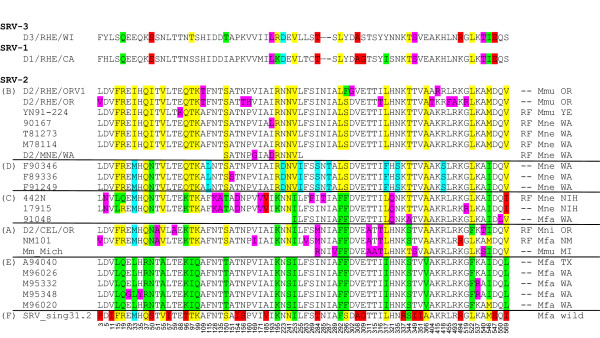
**Alignment of variable amino acid positions within SRV-2 *env *sequences**. This column alignment presents only those amino acid positions that vary in one or more of the twenty-one SRV-2 *env *sequences analyzed; exact position within the complete *env *sequence (Figure 2) is indicated at the bottom of each column. The analogous amino acid positions of the closely related SRV-1 and SRV-3 sequences are shown for comparison. The macaque species, origin and RF status for each SRV-2 isolate are indicated on the right. Colored residues indicate the amino acid groupings upon which the phylogenetic analysis is based. Non-conserved amino acid variants are shaded (magenta). (Mne) *Macaca nemestrina*, (Mni) *Macaca nigra*, (Mfa) *Macaca fascicularis*, (Mmu) *Macaca mulatta*.

Pairwise comparisons of the different subtype *env *sequences revealed amino acid conservations ranging from 96.7% between subtypes A and E and between subtypes B and D, to 93.6% between subtypes E and F (Table 2). Seventy-two amino acid positions (13% of the entire sequence) showed differences in at least one of the sequences analyzed, while fifty-five of these differences occurred in more than one of the sequences. A comparison using the seventy-two variant positions visually demonstrated the basis of the sequence differences between the six subtypes (Figure 3). Some of these sequence differences were conserved within a specific subtype. For example, at amino acid position 33 an isoleucine (I) was conserved in all of the SRV-2B isolates, while a leucine (L) was conserved in all of the SRV-2E isolates (Figure 3). On the other hand, some amino acids were conserved across subtypes, as seen in the SRV-2A, SRV-2C, SRV-2D, and SRV-2F sequences which all contained a methionine (M) at aa33. Frequently, there were single amino acid differences in one isolate within a subtype which were not conserved in the other isolate sequences, i.e. glycine (G) at aa29 in the M95348 isolate of subtype SRV-2E. Due to the fact that in most cases PCR amplification products were sequenced directly, without cloning, these differences would not reflect Taq polymerase errors.

### Genetic variation of SRV-2 within an individual infected macaque

In order to determine the genetic variation of SRV-2 within the same animal, multiple clones of a PCR amplification product encoding a 439 aa fragment of the *env *gene were characterized from two different macaques (F91249, T82422). Eight different clones were obtained from each animal. Sequence analysis of each clone revealed random nucleotide differences between each cloned DNA within an animal (data not shown). However, no nucleotide difference occurred in more than one clone, suggesting that the observed differences were the result of errors induced by Taq polymerase during the PCR amplification step. These data revealed no evidence for the presence of multiple strains of SRV-2 within a single individual.

We further analyzed the genetic variation within an SRV-2 strain from an individual macaque over time. Two tissue samples from macaque M95332 which contained an SRV-2E subtype had been collected at two time points six years apart, thus spanning the evolution of this virus over the time frame from 1997–2003. The complete *env *gene was amplified from each of these samples and compared. No differences were detected between the two sequences. Furthermore, we compared the isolate in M95332 with the isolates from two animals in the same cohort, M96020 and M96026, and the mother of M96026, A94040, who presumably introduced this SRV-2E isolate into the Washington NPRC colony nine years before the last M95332 isolate was sequenced. This comparison revealed only one amino acid difference between the sequence of M95332 and those of M96020, M96026 or A94040, showing very little variation within this SRV-2E strain even between different animals.

### Structural conservation of the env gene between different SRV-2 subtypes

The SRV-2 *env *gene encodes a precursor polypeptide that undergoes both glycosylation and proteolytic processing during a maturation process that results in the expression of the mature membrane-bound glycoprotein integrated within the virion envelope. The envelope glycoprotein interacts with host cellular receptors to initiate virus adsorption and penetration, and plays an important role in determining cell and tissue tropism. Thus, sequence variation in the *env *gene can ultimately affect or determine the pathogenic potential of the virus. Interestingly, our data showed that 87% of the amino acids within the 574 amino acid *env *sequence exhibited no variation in the different SRV-2 isolates examined (Figures 2 and 3). Within the variant amino acid positions, in most cases, only conservative changes were identified. The conservative nature of the *env *gene variability was further highlighted by the finding that, in many of the positions, the variant amino acid in the SRV-2 isolate was an amino acid found in either or both of the more distantly related SRV-1 or SRV-3 serogroup prototypes. For example, leucine (L) at aa193 was found only in one SRV-2B subtype isolate but in both SRV-1 and SRV-3 prototypes. While for the most part the variant positions were scattered evenly throughout the *env *sequence, several highly conserved regions were identified. The N-terminal region aa60-aa95 was completely conserved between all SRV-2 isolates examined (Figure 2).

Interestingly, this region was quite distinct from the homologous regions in SRV-1 and SRV-3. The C-terminal region from the putative N-linked glycosylation site at aa345 to the C-terminus was extremely well conserved among the SRV-2 isolates with only an occasional amino acid variant. This conserved region within the SRV-2 isolates is homologous to regions within the SRV-3 gp22/20 protein where several domains have been studied in detail. Such domains include the gp70/gp20 proteolytic site (aa382) [25], the known fusion domain (aa384-aa410) [26], the heptad repeat region (aa409-aa462) [27], the immunosuppressive peptide (aa443-aa477) [25,28], a membrane spanning domain (aa516-532) [15], and the region spanning the gp22 processing site (aa558) [25](see Figure 2). In contrast to the conserved N-terminal region, the C-terminal region was highly conserved not only between SRV-2 subtypes but also with the SRV-1 and SRV-3 sequences, with one region (aa419-aa485) completely conserved between the isolates from the different SRV serogroups. All twenty-two cysteine (C) residues were conserved between SRV-2 isolates and the SRV-1 and SRV-3 prototypes (Figure 3) indicating that all SRV serogroups maintain the same basic disulfide-linked three-dimensional *env *structure. SRV-1 and SRV-3 sequences contained three additional conserved cysteine residues which were not found in the SRV-2 isolates. Eleven putative N-linked glycosylation sites (NXS/T) were conserved in all the SRV-2 isolates and the SRV-1 and SRV-3 prototypes.

### Sequence and structural conservation of an immunosuppressive peptide

**Figure 4:**
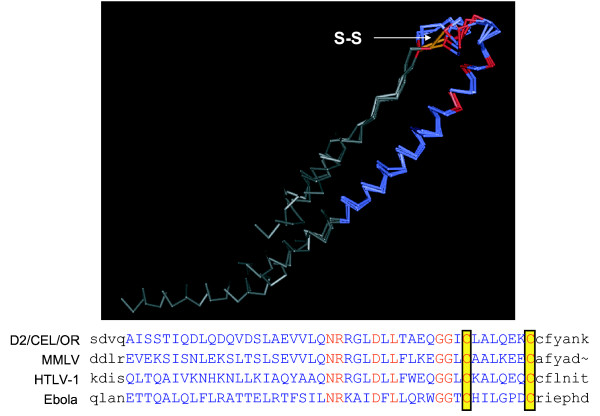
**Structural and sequence alignment of the highly conserved putative disulfide-bonded loop region of the SRV-2 *env *and structurally similar regions of other viral envelope proteins**. Structural similarities to the SRV-2 *env *protein were identified by querying the NCBI protein structure database using 3D-PSSM and Cn3D. A region within the C-terminal domain of SRV-2 *env *which was identical in all SRV-2 isolates and SRV-1 and SRV-3 prototypes (aa426-471) was predicted to have structural similarities to a disulfide-bonded loop presumed to be important for virus-cell fusion in a number of RNA viruses and retroviruses, including Ebola virus 1: 1EBO_A (Gp2); Ebola virus 2: 2EBO_A (Gp2); MMLV (Moloney murine leukemia virus): 1MOF (coat protein); HTLV-1 (human T-lymphotropic virus): 1MG1_A (gp21) (see text). The disulfide bridge is indicated by S-S.

To further explore the conservation detected in the C-terminal region of the different SRV-2 subtypes and the SRV-1 and SRV-3 serotypes, we searched the existing structural databases for similar three-dimensional structures with 3D-PSSM [29] using the D2/CEL/OR subtype 2A sequence as probe. 3D-PSSM is a program that uses a threading algorithm to map the input sequence onto known 3-dimensional structures based on several parameters including amino acid sequence, multiple alignments, and secondary structure predictions. A region of the SRV-2 *env *polypeptide between amino acids 420–485 that was completely conserved in all SRV-2 isolates and in the SRV-1 and 3 prototypes was found to have strong structural homology to the C-terminal domains in the envelope proteins of two other retroviruses, Moloney murine leukemia virus (MMLV) and human T-lymphotropic virus (HTLV-1), as well as to a domain in the envelope protein of Ebola virus, a ssRNA virus. The known crystal structures of the MMLV, HTLV-1 and Ebola envelope proteins revealed that these domains form a highly conserved hairpin loop structure stabilized by a disulfide bond [30-32]. This loop structure is believed to be responsible for viral fusion with cellular membranes in several virus species, some of which share little or no obvious evolutionary relationship. Such viruses include the above mentioned oncogenic retroviruses, the orthomyxovirus influenza [33], the lentiviruses HIV-1 and SIV [34,35], the paramyxovirus SV5 [36,37], and filoviruses [38]. Three-dimensional structural predictions, using the Cn3D structure viewing program, revealed an almost perfect alignment between the structure predicted for the SRV-2 domain and the known crystal structures of the other proteins, even though only nine amino acids in a 45 aa stretch were conserved (Figure 4). The region corresponding to the conserved domain within the SRV-2 isolates has been previously identified as an immunosuppresive peptide in several oncogenic retroviruses capable of inducing an immuosuppressed state in their hosts (FeLV, MuLV, REV-A) [39]. In addition, the entire gp20 protein of SRV-3 has also been found to have immunosuppressive properties [40]. Studies with SRV-3 have revealed that residues in the immunosuppressive peptide found within the gp20 protein subunit are responsible for binding to the gp70 protein subunit and are crucial for virus-cell fusion [25,28].

## Discussion

We investigated the genetic diversity of the serogroup 2 simian retroviruses (SRV-2) in four different wild-caught or captive macaque species from six different primate centers within the US over a 23 year time period. We identified at least six different SRV-2 subtypes by molecular comparison of the complete *env *gene from twenty-two different isolates. Our results indicate that separate introductions of at least six parental virus subtypes have occurred in the captive macaque populations in the U.S. with subsequent horizontal transfer between macaque species and primate centers.

It is most likely that divergent SRV-2 strains were introduced to the United States via importations of different species of infected macaques from different geographical areas. Procurement from common sources, close contact in primate holding facilities, and traffic between primate centers would explain the spread of virus across the captive macaque populations and between macaque species. The introduction of the SRV-2E subtype into the Washington NPRC provides one example for such a virus transfer that was evident from our study. In 1994, a female cynomolgus macaque, A94040, was purchased by the Washington NPRC for breeding purposes. At the time of transfer, this animal was negative for SRV-2 by serology but was later shown to be positive by virus culture. Our analysis of DNA from PBMCs collected in 1997 revealed that A94040 was infected with an SRV-2E subtype, that was not present in other macaques sampled at the Washington NPRC before 1994. The offspring of A94040, M96026, born in 1996, became infected with SRV-2 and analysis of PBMCs collected in 2003 revealed the presence of an SRV-2E isolate identical to that of its mother. Our analysis demonstrated that siblings with the same father as M96026, but a different mother, were infected with SRV-2E isolates that were nearly identical (1–3 aa differences) to that of M96026 and its mother A94040

Our data demonstrates that the *env *gene of SRV-2 is very stable suggesting a remarkable adaptation of the virus to its host. Within the five isolates of SRV-2E obtained from a cohort of cynomolgus macaques at the Washington NPRC, only 0–3 amino acid differences within the 574 aa envelope protein were detected. In addition, we found no evidence for variation of the viral *env *gene within a single individual over a 6 year period. Surprisingly, even viral isolates from different primate centers from different macaque species separated in time by as much as 20 years showed a high degree of conservation. The SRV-2B isolates obtained seven years apart from the rhesus macaque, YN91-224, at the Yerkes NPRC and the pig-tailed macaque, T81273, at the Washington NPRC, differed by only one amino acid. Our data confirm earlier studies which showed a remarkable stability of the SRV-2 genome over time by analysing partial *env *sequences in smaller and more restricted samples [17,41].

The stability of the viral *env *gene over time within any given subtype suggests that the different SRV-2 subtypes evolved in the wild over long periods of time in segregrated primate hosts. Such segregation could be dictated by constraints involving different geographical areas, different niches within the same geographical area, and/or different natural host species. In our study, the natural host species for only one of the SRV-2 subtypes was apparent. The SRV-2F subtype was identified in a number of cynomolgus macaques in the wild on the island of Singapore. Interestingly, the SRV-2F subtype was clearly distinct from all the subtypes present in captive populations of cynomolgus, rhesus, Black celebes, or pig-tailed macaques, suggesting that none of these five subtypes originated from a cynomolgus macaque reservoir in Singapore. To date, no SRV-2 reservoir has been identified in wild-living rhesus macaques which are native to India [42]. Thus, the natural host species of SRV are likely to be found in Southeast Asia. However, further analysis of SRV-2 isolates directly from wild-caught animals is needed to understand the natural reservoirs for these viruses in more detail.

Our initial impetus to study the genetic variation within the SRV-2 serotype was to determine whether there was an association between virus subtype and SAIDS-RF. Our data revealed that SAIDS-RF was associated with three SRV-2 subtypes, 2A, 2B and 2C, in multiple species of macaque, including pig-tailed, rhesus, cynomolgus and Black celebes. A total of eight RF cases were examined from five primate centers including the Washington, Oregon, and Yerkes NPRCs, the NIH primate center and the Lovelace Respiratory Research Institute in New Mexico. While SRV-2A was associated with RF in celebes and cynomolgus macaques, the SRV-2B subtype was associated with RF in pig-tailed and rhesus macaques. The SRV-2C subtype was only associated with RF in pig-tailed macaques. No obvious sequence similarities were detected between the SRV-2A, -2B and -2C subtypes which would correlate with the RF association. In two of the RF cases, RF occurred soon after experimental infection with SIV or SHIV. The rhesus macaque YN91-224 which was infected with an SRV-2B subtype was diagnosed with RF after undergoing an experimental infection with SIV at the Yerkes NPRC (personal communication, H. McClure). The SRV-2C infected pig-tailed macaque 442N was diagnosed with RF 24 weeks after infection with a pathogenic strain of SHIV [24]. Thus, our studies revealed only an association between the SRV-2A subtype and SAIDS-RF in Black celebes macaques and between the SRV-2B subtype and SAIDS-RF in pig-tailed macaques, in the absence of other known immunodeficiency agents.

We have recently identified a single case of RF in a rhesus macaque experimentally infected with a pathogenic strain of SIV [43]. This animal was negative for all SRV serotypes using type-specific qPCR assays. Additionally, four cases of SAIDS-RF were reported in 1983 in a colony of Taiwanese rock macaques at the New England NPRC which were endemically infected with SRV-1 [44]. Similarly, a single case of SAIDS-SF, the subcutaneous form of RF, was reported in 1983 in a colony of rhesus macaques endemically infected with the D1/RHE/CA subtype of SRV-1 at the California NPRC [3]. Even though the vast majority of RF cases in the different macaque colonies were associated with SRV-2 serotypes, these findings suggest a broader role for different SRV serotypes and possibly other retroviruses such as lentiviruses as cofactors in the development of RF, albeit with an apparent low efficiency.

Factors which are likely to play a major role in the development of RF, include differences in the severity and type of immunosuppression caused by an SRV-2 or lentivirus infection, and co-infection with RFHV. Current data suggests that the macaque herpesvirus, RFHV, may play a causative role in the etiology of RF [23,45,46]. We have developed PCR assays to detect the host-specific variants of RFHV in rhesus (RFHVMm) and pig-tailed (RFHVMn) macaques and have identified RFHV in RF lesions from the macaques infected with both SRV-2B (T82422, 90167, M78114, and YN91-224) and SRV-2C (442N) in this study [[45], and unpublished results]. It is not known, at this point, whether the macaque cohorts infected with the SRV-2D, -2E and -2F subtypes which were not associated with RF, were also co-infected with RFHV.

Differences in disease pathology and severity have been observed in SRV-2 infections which could impact RF development. Within a cohort infected with a single SRV-2 subtype, different outcomes have been reported, including a viremic state with rapid progression of SAIDS, a low-grade viremia with a chronic milder form of the disease, and a strong antibody response with no overt signs of disease [47]. Similarly, the same SRV-2 subtype can elicit differences in disease severities in different macaque species. The D2/CEL/OR isolate, for example, caused severe immunodeficiency in Celebes black macaques, but when the same isolate was transmitted to rhesus macaques, the animals seroconverted and remained virus- and symptom-free [13]. Conversely, the D2/RHE/OR isolate caused mild disease in rhesus macaques but severe fatal immunodeficiency disease in Japanese macaques (*Macaca fuscata*). On the other hand, the closely related SRV-2B isolates, D2/RHE/OR and D2/RHE/OR/V1, which differ in only 17 amino acids over their entire genomes, were found to induce vastly different disease outcomes in rhesus macaques and to also display differences in tropism in cell culture assays [15]. The D2/RHE/OR variant was associated with only mild disease while the V1 variant caused severe SAIDS.

In our study, the *env *sequence from the different SRV-2 isolates was highly conserved overall, with 93–96% amino acid identity between isolates from different subtypes and 97–100% amino acid identity between isolates within a subtype. A hypervariable region was detected between aa 284–321 near the C-terminus of the gp70 protein. Even within this variable region, most amino acid changes were conservative or conserved with the SRV-1 or SRV-3 sequences or both, underlining the stability of the *env *protein. Interestingly, all potential *N*-linked glycosylation sites were conserved between the SRV-2 isolates, and even with the related SRV-1 and SRV-3 serotypes. This is in stark contrast to HIV and SIV which exhibit extreme variation in number and precise location of *N*-linked glycosylation sites [48]. Such variation is believed to be a major pathway for immune evasion for lentiviruses. Thus, the strict conservation of glycosylation sites between the various SRV-2 isolates may help explain the high efficiency of neutralizing antibodies against SRV-2 infections [49].

Our analysis showed that 15.5% of the amino acid positions within the receptor-binding surface-exposed (SU) subunit gp70 were variable while only 8.7% of the amino acid positions within the transmembrane (TM) subunit gp22 differed between isolates. Differences were more concentrated in the C-terminal portion of gp70 (last 100 aa) but also affected the N-terminal signal peptide domain, as well as the known B- and T-cell epitopes (aa96-102, aa127-153). The B- and T-cell epitope at aa96-102 is of particular importance since naturally occurring neutralizing antibodies to SRV-2 are directed against this area [50] and it confers binding to the RD114/simian type D retrovirus receptor, a neutral amino acid transporter [51,52]. Three amino acid positions in this seven amino acid domain displayed variations between isolates. We identified three sequence variants within the T-cell epitope at aa127-152, but only one difference in the 2^nd ^T-cell epitope located at aa233-249 [53]. Although some of these differences are of conservative nature, the remaining changes could affect the ability of the virus subtypes to elicit an immune response and lead to differences in disease outcome.

The gp22 subunit was far less variable between isolates than the gp70 subunit which likely is the result of sterical restrictions imposed by a string of conserved functional domains. The TM subunits of most retroviruses, including SRV-3, contain an N-terminal hydrophobic fusion peptide followed by a putative coiled coil-forming sequence, a disulfide-bonded loop connected to a shorter C-terminal alpha helix, a region with one or more N-linked glycosylation sites, a hydrophobic membrane-spanning sequence, and, as in SRVs, a cytoplasmic tail. Absolute sequence conservation between different SRV-2 subtypes and the SRV-1 and SRV-3 serotypes was seen within a region of the C-terminal gp20 domain which has been shown to be crucially important for the interaction between the SU and TM domains and for the fusion of viral and cellular membranes in MPMV. Using a threading algorithm, we showed that the homologous region within SRV-2, between aa426 and aa471, was structurally similar to a region in other retroviruses including MMLV and HTLV-1 [31,32,54] and the filovirus Ebola [38]. In these and other distantly related retroviruses [34,35] as well as in the orthomyxovirus influenza, the conserved disulfide-bonded loop plays a highly pivotal role in stabilizing a chain reversal which provides a hinge-like function that brings the fusion peptide into proximity to the target cell membrane during the fusion process [33]. We propose that SRV-2, in analogy, uses a similar mechanism for host cell membrane fusion.

## Conclusion

In conclusion, our study revealed the presence of five SRV-2 subtypes circulating among four macaque species held in US primate centers. Three of these subtypes were associated with RF in some macaque species, although a correlation between sequence variation and disease could not be established. A sixth and different subtype was found in wild cynomolgous macaques on the island of Singapore suggesting that the subtypes found in captive macaques in the U.S. did not originate from this virus reservoir. Since the viral subtypes discovered in captive monkeys did not display a macaque species specificity, the nature of the original reservoir for these viruses is unclear. Our studies also showed that the envelope protein of SRV-2 was very stable suggesting a high degree of adaptation of SRV-2 to its host. In all likelihood, simian retroviruses have evolved very slowly in geographically distinct macaque species, and only the physical contact with other macaque species in captivity has allowed single virus subtypes to spread between species.

## Methods

### Animals

Tissue samples were obtained from a variety of macaque species naturally infected with SRV-2 that were housed at different primate research centers in the U.S. over the last twenty years. Some of these macaques were asymptomatic, while others had been diagnosed with SAIDS-RF (see Table 1). The tissue samples included RF tissue from RF positive animals, tongue, spleen, tonsils and PBMCs or PBMC/Raji cell co-cultures. Samples had been collected over a wide time-range (1983–2003), starting at the height of the SRV-2 epidemic in 1982–83. Macaque species included pig-tailed macaques (*Macaca nemestrina *(Mne)), Celebes black macaques (*Macaca nigra *(Mni)), cynomolgous macaques (*Macaca fascicularis *(Mfa)) and rhesus macaques (*Macaca mulatta *(Mmu)). Tissue samples were obtained from various primate research centers including the Washington NPRC (Seattle, WA; C.-C. Tsai and M.E. Thouless), Yerkes NPRC (Atlanta, GA; Harold McClure), National Institutes of Health (Bethesda, MD; Riri Shibata), Lovelace Respiratory Research Center (Albuquerque, NM; Carole Emerson), University of Michigan (Ann Arbor, MI; Nina Woodford); as well as one wild cynomolgous macaque whose blood was drawn during a catch-and-release process in the forests of Singapore (University of Washington; Lisa Jones-Engel).

### DNA isolation

Frozen tissue samples or cell pellets were quickly thawed in a standard proteinase K extraction buffer containing 0.1% SDS and vortexed. Paraffin-embedded formalin fixed samples were first treated with xylenol to remove the paraffin before extraction. Samples were digested over night at 50°C and DNA was isolated by standard phenol/chloroform extraction and ethanol precipitation.

### PCR amplification, cloning and sequencing of the SRV-2 *env *genes

To obtain the complete sequence of the different SRV-2 *env *genes, template DNA was amplified by PCR using SRV-2 specific primers. Primers *srv2-env a *(5'-CCTGAGATCACTCCTTTTCTTTGCTCAT-3') and *srv2-env1 b *(5'-CCGTCATTGGCTGACCAGTTTAG-3') were used to obtain a 1,796 bp PCR product containing the complete SRV-2 *env *sequence. In some cases, the primers *srv2-env a *and *srv2-env b *(5'-CAGTTGAGACGGCAGTGGTT-3') were used to obtain a 1,235 bp fragment which overlapped with a 1,041 bp fragment obtained using *srv2-env1 a *(5'-GAGTGCTGGCTATGCTTACCATC-3') and *srv2-env1 b*. PCR fragments were purified with the GeneClean III Kit (Q-Biogen) and directly used as templates in a sequencing reaction.

To search for SRV-2 variants in the same animal, a 439 bp fragment of the *env *gene was PCR-amplified from genomic DNA of two macaques (T82422, F91249) using Platinum Taq polymerase and the primers: *SRV2-env 1a *(5'-GAGTGCTGGCTATGCTTACCATC-3') and *SRV2-env b *(5'-CAGTTGAGACGGCAGTGGTT-3'). The fragments were subcloned into the pCR-Blunt vector (Invitrogen Life Technologies, Carlsbad CA) using standard procedures. Eight clones were obtained from each animal for sequence analysis.

Sequencing was performed on an ABI model 310 automated sequencer with Prism Big Dye terminator cycle sequencing ready reaction kit with Amplitaq DNA polymerase FS (Applied Biosystems). DNA sequences were analysed using Sequencher 4.1.4 (GeneCodes).

### Sequence analysis

Multiple nucleotide and encoded amino acid alignments were done with Vector NTI 9.0.0 (InforMax) based on the ClustalW algorithm (EMBL, Heidelberg, Germany) or with the ClustalW program [55] directly. The location of the signal peptide was determined by SignalP 3.0 [56], SigCleave [57], and iPSORT [58]. The location of transmembrane domains was determined with MEMSAT2 [59] and Tmpred [60]. Putative glycosylation sites were determined with NetNGLyc 1.0 [61].

### Three-dimensional structure analysis

The program 3D-PSSM [62] was used to identify proteins with structural similarities to the SRV2 *env *prototype (D2/CEL/OR). The structures for the six highest scoring proteins were obtained from the Molecular Modeling Database (MMDB)[63] which contains experimentally determined three-dimensional biomolecular structures obtained from the Protein Data Bank. Matching structures were aligned with SRV2 *env *using NCBI's 3D-structure viewer Cn3D v4.1 [64]. Using NCBI's vector alignment search tool (VAST)[65], we determined further protein structure neighbors by direct comparison of 3-dimensional protein structures stored in MMDB. This allowed us to further evaluate possible structural and functional domains of the SRV2 *env *protein.

### Phylogenetic analysis

Multiple sequence alignments were performed with ClustalW [55]. Phylogenetic analysis of amino acid sequences was done with protein maximum-likelihood (ProML) method from the PHYLIP package version 3.62 of phylogenetic analysis programs (.)[66] (University of Washington, Seattle). Bootstrap analysis was performed using the programs Seqboot and Consense from the PHYLIP package. The treefiles were displayed with TreeView [67]. Identical tree topologies were obtained by using neighbor-joining analysis of the protein distance matrices and by a parsimony method as implemented in the PHYLIP package. All of the major branch points were strongly supported by boot-strap analysis and statistical evaluations within ProML.

## Competing interests

The author(s) declare that they have no competing interests.

## Authors' contributions

JP-S carried out the sequence analysis, the three-dimensional structure analysis, the phylogenetic analysis, as well as the cloning studies, participated in the PCR and cloning efforts, and drafted the manuscript. TM participated in the PCR and cloning efforts. AGB developed the PCR cloning approach and participated in the PCR and cloning efforts. RG provided the SRV-2 envelope sequence from the wild-caught macaque, MET supplied DNA samples and participated in the design and coordination of the study. C.C-T provided a large number of RF and non-RF tissue samples from his study collection. TMR conceived of the study, and participated in its design and coordination and helped to draft the manuscript. All authors read and approved the final manuscript.

## Figures and Tables

**Table 1:** Macaque sources of SRV-2 isolates

**SRV Serogroup/Subtype**	**Animal/Virus Isolate**	**Species**^**1**^	**Origin**^**2**^	**RF**^**3**^	**Sample**	**Date**^**4**^	**Comments**
SRV-1	RM 18610/ D1/RHE/CA	Mmu	CA/cb	-	Tissue homegenate/ in vivo passage	1983	[Genbank:M11841] [6]
SRV-3	D3/RHE/WI	Mmu	WI	-	Breast tumor	1970	Mason-Pfizer monkey virus (MPMV) [Genbank:M12349] [2]
SRV-2A	D2/CEL/OR	Mni	OR	**+**	PBMC/Raji culture	1985	[Genbank:M16605] [14]
-2A	Mm_Mich	Mmu	MI	-	Spleen	1997	
-2A	NM101	Mfa	NM/cb	**+**	Tongue	2004	
SRV-2B	D2/RHE/ORV1	Mmu	OR	**-**	PBMC/Raji culture	1989	[Genbank:AF126468] [15] → severe SAIDS in rhesus
-2B	D2/RHE/OR	Mmu	OR	**-**	PBMC/Raji culture	1986	[Genbank:AF126467] [16] → mild SAIDS in rhesus
-2B	YN91-224	Mmu	Yerkes/cb	**+**	RF tumor	1991	Experimentally infected with SIV in 1989
-2B	90167	Mne	WA/tr	**+**	RF tumor	1995	
-2B	T82422	Mne	WA/cb	**+**	RF tumor	1984	Diagnosed with RF/SF^5^
-2B	M78114	Mne	WA/cb	**+**	RF/SF tumor	1984	Diagnosed with RF/SF [68]
-2B	D2/MNE/WA	Mne	WA/cb	**+**	RF tumor	1982	[17]
SRV-2C	442N	Mne	NIH/tr	**+**	RF tumor	1996	Experimentally infected with SHIV in 1996 [24]
-2C	17915	Mne	NIH/tr	**-**	PBMC	1996	
-2C	91048	Mfa	WA/tr	-	PBMC	1997	
SRV-2D	F90346	Mne	WA/cb	**-**	PBMC	1992	Same parents as F91249
-2D	F89336	Mne	WA/cb	**-**	PBMC/A549 culture	1994	Same father as F91249 and F90346
-2D	F91249	Mne	WA/cb	**-**	PBMC/A549 culture	1991	Same parents as F90346
SRV -2E	A94040	Mfa	WA/tr	**-**	PBMC	1997	Transferred to WA in 1994, SRV-2 positive, mother of M96026
-2E	M95332	Mfa	WA/cb	**-**	PBMC Spleen	1997 2003	
-2E	M95348	Mfa	WA/cb	**-**	PBMC	2003	Same father as M96020 and M96026
-2E	M96020	Mfa	WA/cb	**-**	Tonsils	2003	Same father as M95348 and M96026
-2E	M96026	Mfa	WA/cb	**-**	PBMC	2003	Child of A94040; same father as M95348 and M96020
SRV-2F	SRV_sing31.2	Mfa	Singapore/wc	-	PBMC	2003	Sampled in wild

^1^Species of macaque from which the sample was taken. Mne = *Macaca nemestrina*; Mmu = *Macaca mulatta*; Mfa = *Macaca fascicularis*; Mni = *Macaca nigra;*

^2^Primate center origin: WA = Washington NPRC; Yerkes = Yerkes NPRC; OR = Oregon NPRC; NIH = National Institutes of Health, Bethesda MD; MI = University of Michigan; NM = Lovelace Respiratory Research Institute, New Mexico; Singapore = sampled in the wild on the island of Singapore; wc = wild caught; cb = colony born; tr = transferred

^3^RF = diagnosed with retroperitoneal fibromatosis

^4^Date = approximate date sample obtained

^5^RF/SF = diagnosed with retroperitoneal and subcutaneous fibromatosis

**Table 2:** *Env *sequence comparison of the SRV2-subtypes

SRV-2 Subtype	Within subtype	Between subtypes^1^				
		
		2B	2C	2D	2E	2F
2A	97.3–98.6%	96.2%	96.3%	96.5%	96.7%	94.8%
2B	98.3–100%	-	94.9%	96.7%	94.8%	94.3%
2C	98.5–99.4%	-	-	95.5%	96.3%	94.3%
2D	99.0–99.8%	-	-	-	95.3%	94.1%
2E	99.1–99.8%	-	-	-	-	93.6%
2F	98.8–100%	-	-	-	-	-

^1^The prototypes of each SRV-2 subtype, as indicated in the legend to Figure 2, were compared
